# Clearance Systems in the Brain, From Structure to Function

**DOI:** 10.3389/fncel.2021.729706

**Published:** 2022-01-31

**Authors:** Jiachen Liu, Yunzhi Guo, Chengyue Zhang, Yang Zeng, Yongqi Luo, Gaiqing Wang

**Affiliations:** ^1^Xiangya Medical College of Central South University, Changsha, China; ^2^Shanxi Medical University, Taiyuan, China; ^3^Department of Neurology, Affiliated Sanya Central Hospital of Hainan Medical University, Sanya, China

**Keywords:** brain waste clearance, glymphatic system, meningeal lymphatic vessels, blood-brain barrier, neuroglia

## Abstract

As the most metabolically active organ in the body, there is a recognized need for pathways that remove waste proteins and neurotoxins from the brain. Previous research has indicated potential associations between the clearance system in the brain and the pathological conditions of the central nervous system (CNS), due to its importance, which has attracted considerable attention recently. In the last decade, studies of the clearance system have been restricted to the glymphatic system. However, removal of toxic and catabolic waste by-products cannot be completed independently by the glymphatic system, while no known research or article has focused on a comprehensive overview of the structure and function of the clearance system. This thesis addresses a neglected aspect of linkage between the structural composition and main components as well as the role of neural cells throughout the clearance system, which found evidence that the components of CNS including the glymphatic system and the meningeal lymphatic system interact with a neural cell, such as astrocytes and microglia, to carry out vital clearance functions. As a result of this evidence that can contribute to a better understanding of the clearance system, suggestions were identified for further clinical intervention development of severe conditions caused by the accumulation of metabolic waste products and neurotoxins in the brain, such as Alzheimer’s disease (AD) and Parkinson’s disease (PD).

## Introduction

**FIGURE 1:**
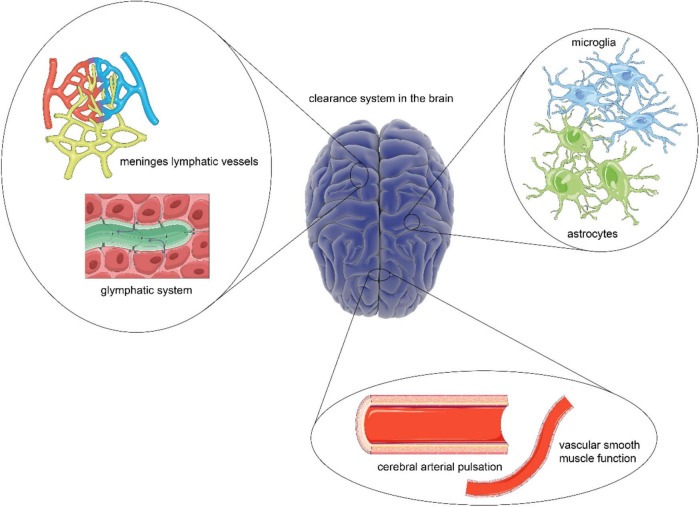
A comprehensive overview of the clearance system in the brain. As the scarcity of lymphoid tissue, the brain has a unique clearance system to eliminate metabolic waste. The construction of a framework based on the glymphatic system and meningeal lymphatic vessels provides a platform for cerebrospinal fluid (CSF) flow, which is essential for the exchange and transport of metabolic waste. Besides, the driving force for CSF flow is provided by cerebral arterial pulsation and smooth muscle function. While the steady states of the process are guaranteed by astrocytes and microglia.

It has previously been observed that despite a high metabolic rate, the brain lacks an actual lymphatic system that aids in removing metabolic waste and toxic agents from the brain (Kumar et al., 2019). Therefore, the clearance system in the brain has long been a subject of great interest in a wide range of neuroscience fields since it was discovered in the 1960s (Bedussi et al., 2015). In recent years, there has been growing recognition of the vital links between the clearance system and disease of the central nervous system (CNS). Therefore, there is an urgent need to have a comprehensive understanding of the clearance system (Zhang et al., 2019). So far, however, the whole clearance system, including the structural characterization and a possible connection between them, has received scant attention in the research literature. Some of the previous studies have generally been focused on analyzing the glymphatic system (Jessen et al., 2015). To some extent, the relationship between other important structures in the brain and the glymphatic system needs further investigation to provide robust evidence for promising clinical intervention based on the clearance system in brain disorders due to the accumulation of waste products (Sun et al., 2018). This article seeks to remedy these problems and propose a theoretical framework based on the clearance system in the brain by systematically reviewing the literature of main structural components, including nerve cells and extracellular vesicles (EVs) at present (Figure 1). The viewpoint presented in this review is to explore the overall structure and function of the clearance system, which will shed new light on future research that will lead to the further potential development of preventive and therapeutic interventions of brain disorders mentioned earlier.

## Structural Basis of Clearance System in the Brain

The proper functioning of the clearance system in the brain requires a stable structure. Also, it has been reported that the glymphatic and meningeal lymphatic have brain lymphoid function. Thus, the “Glymphatic system” section will establish the framework of the structure, function, and interactions of two systems, which contributes to a better understanding of physiological and pathological conditions of the clearance system in the brain.

## Glymphatic System

The first pioneering study in 2012 identified a brain-wide pathway in mice with small fluorescent tracers and termed it as a “glymphatic” system (Iliff et al., 2012). With the development of the diffusion MR technique and diffusion tensor imaging (DTI), subsequent researches indicated the presence of the glymphatic system in the human brain (Taoka et al., 2017; Yokota et al., 2019). The glymphatic system supports interstitial solute and cerebrospinal fluid (CSF) clearance from the brain and shares functions with the lymphatic vessels.

**FIGURE 2:**
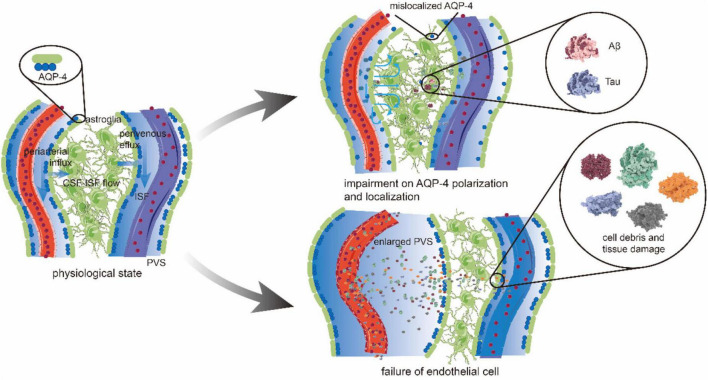
The glymphatic pathway and pathological changes. CSF in periarterial spaces flows across the glial basement membrane and astroglial endfeet to enter the brain parenchyma. The aquaporin 4 (AQP4) is highly expressed on the endfeet of astrocytes, forming the outer wall of the perivascular space (PVS) and facilitating the CSF influx into the interstitium. After mixing with interstitial fluid (ISF) and waste metabolites, CSF is transported toward to perivenous space in a polarized net fluid movement. Ultimately, CSF exits along the lymphatic vessels and arachnoid granulations for the removement of solutes from the brain parenchyma accumulated during neural activity. Under pathological conditions, the brain may have an impairment on AQP4 polarization and localization. In patients with Alzheimer’s disease (AD), AQP4 depolarization leads to the reduction in CSF influx from periarterial space. At the same time, the direction of CSF-ISF flow is disrupted or even reversed, which reduces the ability and efficiency to transport amyloid β (Aβ) protein. The concentration of Aβ increases gradually, and deposition occurs in the brain parenchyma and periarterial space. Vascular pathology causes endothelial cell (EC) failure, producing the cell debris and protein deposition that gives rise to the obstruction and inflammation of PVS. Increased PVS fluid and the abovementioned factors causes enlarged PVS, which then leads to a further accumulation of toxic metabolic by-products, such as Aβ and tau.

The glymphatic system is a brain-wide route that comprehends the entrance, interstitial movement and exchange, and exit of CSF. There are several critical components in this system, including CSF, interstitial fluid (ISF), perivascular space (PVS), cerebral vascular, glial cells, and the astrocyte aquaporin 4 (AQP4)-controlled water channels. Produced by the choroid plexus, CSF flows through the lateral ventricles, the third ventricles, and the fourth ventricles into the subarachnoid compartment connected to the PVS (Iliff et al., 2012; Nedergaard and Goldman, 2020). Cerebrospinal fluid is then driven into the PVS under the pulsatility of the penetrating arteries (Benveniste et al., 2019). PVS is an open, low-resistance space formed by the vascular endfeet of the astrocyte, which serves as a wall, strengthening the entire cerebral vascular bed (Nedergaard and Goldman, 2020). Therefore, CSF can be considered as river-carrying sediment, which is metabolic waste, and PVS is the conduit where CSF flows (Benveniste et al., 2019). AQP4 is one of the subtypes of AQP that are highly expressed in the endfeet of the astrocyte. Working similar to sluice gates, AQP4 allows CSF to enter the brain parenchyma to exchange metabolites (Rasmussen et al., 2018). Interstitial fluid takes up 12%–20% of the fluid compartments in the brain, and it mixes with the CSF influx within the tissue toward the perivenous spaces (Benveniste et al., 2019). Eventually, the CSF-ISF fluid and small-size and hydrophilic waste drain to meningeal lymphatic vessels (MLVs) and the blood circulation (Figure 2).

Having discussed how to construct the glymphatic system, it is essential to probe into the significant roles in neurophysiology (Plog and Nedergaard, 2018). The function of the glymphatic system in waste clearance includes three aspects: (1) Since the PVS serves as a conduit sink for CSF/ISF movement, the glymphatic system constructs a trans-arteriovenous network in transmitting hydrophilic waste, such as lactate (Lundgaard et al., 2017). (2) The perivascular influx of CSF is involved in the clearance of macromolecules that are then absorbed by the downstream lymphatic network, forming a “front end” of the glymphatic-lymphatic connection (Da Mesquita et al., 2018b; Benveniste et al., 2019). (3) It serves complementary roles with the blood-brain barrier (BBB) in providing a well-conditioned neuronal environment in the prevention of mechanical disruption and waste accumulation (Verheggen et al., 2018).

With multiple critical roles in CNS physiology, it is not difficult to comprehend that the lesions of the major structures of the glymphatic system can weaken its capabilities to remove waste and have a devastating impact on brain health. For example, the depolarization, mislocalization, and deletion of AQP4 in the aging brain are associated with the impairment of perivascular CSF recirculation, which may together contribute to the accumulation of amyloid β (Aβ) protein and Tau proteins in the brain (Kress et al., 2014; Zeppenfeld et al., 2017) (Figure 2). A hypothesis might be further explained as follows: due to a loss of AQP4 localization, the extracellular fluid containing Aβ, which should have proceeded on to the perivenous spaces, is now attenuated in the brain interstitial space and flows disorderly (Jessen et al., 2015; Reeves et al., 2020). Also, it results in the elevated extracellular concentration of the protein (Nedergaard and Goldman, 2020). Given the combination of other factors, such as shear stress, ionic strength, and local pH, waste accumulation comes into existence (Burke et al., 2013). A recent study that used pharmacological blockade to perturb AQP4 polarization in rTg4510 mice has observed an ∼85% reduction in MRI-quantified CSF-ISF exchange and a similar decrease in tau clearance from the brain (Harrison et al., 2020), which is consistent with what was mentioned earlier. It suggests that AQP4 may serve as a significant predictor for the status of certain diseases, including chronic sleep disruption (Zhang et al., 2020), Alzheimer’s disease (AD) (Zeppenfeld et al., 2017), and traumatic brain injury (TBI) (Piantino et al., 2019), whose pathogeneses are all related to the inefficient waste removal process. To further discuss the imaging progress of AQP4 detection, it is known that the diffusion methods can evaluate the molecular dynamics of water in brain tissue by the signal changes of motion-probing gradients (Taoka and Naganawa, 2020). Diffusion tensor imaging analysis along the PVS (DTI-ALPS) has the advantage of being non-invasive. It is applied to measure the motion of water molecules along the PVS direction in recent studies, indicating that diffusion MRI can evaluate the CSF-ISF exchange (Harrison et al., 2018; Taoka, 2021). The expression of AQP4 plays an essential role in CSF-ISF exchange, and the apparent diffusion coefficient (ADC) can be regarded as a biomarker of AQP gene expression (Ohene et al., 2019), suggesting that diffusion MRI can monitor the changes of AQP4 molecules while evaluating the glymphatic system. What is more, a study of Sindex and shifted water diffusion coefficient (sADC), i.e., the two biomarkers that are more sensitive to tissue microstructure than ADC, further makes it possible to monitor the expression of AQP4 *in vivo* (Debacker et al., 2020).

The PVS is the space filled with CSF-like fluid, which follows penetrating vessels (Iliff et al., 2012; Mestre et al., 2017). The pulsatility of the penetrating arteries drives CSF into the neuropil along with the periarterial spaces (Aldea et al., 2019; Sloots et al., 2020). Typically, vascular cell debris, particulate, and protein deposition can cause the enlargement of PVS (Brown et al., 2018), but the driving forces within the glymphatic system do not change synchronously (Figure 2). Therefore, it is reasonable to work on the principle that enlarged perivascular space (ePVS) might be the pathological changes that occur early in minor vessel diseases (Brown et al., 2018; Wardlaw et al., 2020), in which secondary neuroinflammation is often observed. In addition to the associations between ePVS burden and minor vessel diseases, mounting evidence also suggests that ePVS may be modulated by sleep and TBI (Opel et al., 2019), revealing the presence of ePVS as a potential state marker for impaired clearance. While researchers have recently started to assess the role of astroglial biology in ePVS (Boespflug et al., 2018), more studies are needed to confirm the relationship between the pathological states of AQP4 and ePVS. Previous studies have been carried out on AQP4 dysfunction and its role in waste accumulation and disease, which may neglect its interrelation and interaction with other components and cells in the glymphatic system. Further study is necessary to uncover their close connection to the waste clearance process. Thus, new insights can be given to discover drugs and therapeutics for neurodegenerative diseases that involve multiple pathological changes.

## Meningeal Lymphatic Vessels

The first serious discussions and analyses of MLVs emerged during the 1800s with Paolo Mascagni (Lecco, 1953). So far, evidence of MLVs was obtained at autopsy (Visanji et al., 2018) that at the level of the superior sagittal sinus, there were lymphatic vessels in the human dura mater. In 2017, Absinta et al. (2017) visualized the lymphatic vessels in the dura mater by brain MRI (Absinta et al., 2017) which innovatively and concretely demonstrates the existence of MLVs.

The MLVs were initially considered mainly residing in the base of the skull (Bakker et al., 2016). The development of MRI has enabled a more delicate and precise structure of MLVs. Through the 3D-rendering of subtraction MRI images, dural lymphatics are discerned running parallel to the dural venous sinuses and along with the branches of the middle meningeal artery (Absinta et al., 2017). Besides, arteries, veins, and cranial nerves draining the contents into the deep cervical lymph nodes (dCLNs) were also detected by MRI (Bakker et al., 2016; Ha et al., 2018), which includes immune cells, CSF, and ISF from the subarachnoid space. Accordingly, MLVs are the downstream drainage of soluble and cellular components in CSF. In T2-fluid attenuated inversion recovery (FLAIR) MRI, lymphatic of the CSF was observed outflow through the jugular foramen into the cisterna magna. Also, most of it flew to the basal MLVs, rather than dorsal MLVs, and reached dCLNs from base MLVs (Ahn et al., 2019).

Similar to the initial lymphatic vessels in anatomy and molecular characteristics, the MLVs express all the traditional markers of endothelial cells (ECs) of the lymphatic vessels, without smooth muscle cells and valves (Louveau et al., 2015). While, a potential lymphatic valve has been detected at the bottom of the skull (Ahn et al., 2019), suggesting that these lymphatic vessels transition from the initial vessel to the collection vessel. Moreover, the diameter of MLVs is smaller than peripheral lymphatic vessels (Louveau et al., 2015).

A way to remove cellular debris and toxic molecules, such as Aβ peptides in the brain, is that CSF influx and ISF efflux through the paravascular pathway (Iliff et al., 2012; Peng et al., 2016), as previously demonstrated. As far as pathological changes are concerned, MLV disorders can impair the efflux of substantial/ISF macromolecules and their drainage to dCLNs, which functionally links the meningeal lymphatics with the CSF influx/ISF efflux mechanism (Da Mesquita et al., 2018b).

As the functions of CSF and ISF in the glymphatic system mentioned earlier, it is speculated that the glymphatic system and the meningeal lymphatic system are inextricably linked in exerting immune and clean functions. CSF, ISF, and meningeal lymphatic flows can be regarded as a holistic model when exploring the mechanism of brain immune surveillance and waste removal. MLVs were proved to be located downstream of the glymphatic system using MRI (Zhou et al., 2020), and rather than dorsal MLVs, basal MLVs are the main pathways to uptake and clear macromolecule and could be hot spots for the drainage of CSF and ISF (Ahn et al., 2019). Researches have shown that injected brain-derived antigen efflux by CSF through MLVs can trigger T cell immune response after passing through dCLNs (Noé and Marchi, 2019). In comparison, the impairment of CSF-ISF exchange leads to reduced clearance of tracers in the brain and damage of drainage to dCLN (Iliff et al., 2012).

The regular operation of the CNS of the brain has strict requirements for the brain environment. Therefore, when the MLVs are damaged or CNS changes, it will affect the function. Recent evidence from animal studies demonstrates that impaired meningeal lymphatic drainage function caused spatial learning and memory impairment (Wang et al., 2019). Additionally, mice with the long-term ablation of MLVs show abnormal gene expression in the hippocampus related to neurodegenerative diseases (Lundgaard et al., 2017), which all suggest that neurodegenerative diseases might be related to meningeal lymphatic disorders. As mentioned earlier, the MLV is an immune tissue structure closely related to CSF. The impaired function of the MLV may affect the removal of metabolic waste in CSF, providing new possibilities for the preventive treatment of certain elder brain diseases manifested as cognitive impairment.

Alzheimer’s disease has been taken as an example, which is one of the common neurodegenerative diseases. We inferred earlier that the obstruction of Aβ precipitation removal may lead to AD. As previously mentioned, the drainage of MLVs is one of the pathways for Aβ clearance, so it is inferred that the damage of meningeal lymphatics is related to AD. Recent observations suggest that the decline in meningeal lymphatic function during aging may aggravate the pathological state of the brain and meningeal amyloid (Ma et al., 2017). It is hypothesized that impaired meningeal lymphatic drainage of CSF will affect Aβ clearance, thus increasing the amyloid load of the brain (Da Mesquita et al., 2018a). Since vascular endothelial growth factor C (VEGF-C) can regulate the development of MLVs, promoting the clearance of amyloid (Wang et al., 2019) makes hope for a novel therapy for elderly AD.

Previous studies have confirmed CSF efflux movement from the brain parenchyma to downstream lymphatic circulation (Iliff et al., 2012). With new studies on the function of the glymphatic system and MLVs emerging (Louveau et al., 2017), it is significant to understand their interaction in detail.

Based on former research, the connection between the glymphatic system and MLVs was demonstrated with fluorescent tracer and dye in 2015 (Louveau et al., 2015). It is found that the MLVs could absorb the fluid from the glymphatic system and transport it into dCLN (Aspelund et al., 2015). Anatomically, Aspelund et al. indicated that the waste products might gain access to the lymphatic vessels through the adjacent subarachnoid space and drainage veins merging into the dura mater (Aspelund et al., 2015). The meningeal lymphangiogenesis was found coupled with the enhanced glymphatic influx under chronically implanted electroencephalography electrodes, which confirmed a close association between glymphatic activity and the meningeal lymphatic vasculature (Hauglund et al., 2020). When devastating cerebrovascular events, such as subarachnoid hemorrhage occurred, a decrease of meningeal lymphatic drainage and the depolarization of APQ4 were observed at the same time (Pu et al., 2019), suggesting a pathological link under the glymphatic-lymphatic connection. In conclusion, the function and dysfunction of MLVs and their relationship to the glymphatic system shed light on a hypothesis that the two systems work as a whole clearance system in the brain. Still, the hypothesis needs to be validated and strengthened by future studies.

## Components and Barrier Protection of Clearance System in the Brain

Based on the complete structure, maintaining the normal biological function of the clearance system in the brain still requires that waste capture and directional movement and barrier protective effects of the process ensure metabolic homeostasis in the brain. A more detailed account of components and barrier protection, including drivers and impact factors, is given in the following section.

## Cerebrospinal Fluid

Produced by the choroid plexus, CSF fills the brain ventricles and the subarachnoid space and surrounds the spinal cord in the adult human brain (Benveniste et al., 2017; Marques et al., 2017). Cerebrospinal fluid flows are a prerequisite for a clearance system to maintain normal biological function. Therefore, exploring the effect of the driving force is required to construct a framework for a complete system (Benveniste et al., 2017). The flow direction of CSF along the intracranial artery was confirmed according to the studies mentioned earlier, which implies the importance of the pulsation of cerebral arteries, such as leptomeningeal arteries, to the driving force (Ringstad et al., 2017). Further observations relating to the flow velocity in CSF confirmed the pulsatile flow of CSF that matches the cerebral arterial pulse (Mestre et al., 2018). Subsequently, more and more evidence has indicated that the pulsatile flow in the arterial wall is a significant driver of the CSF (Taoka and Naganawa, 2018). However, as research in the fields of the clearance system surges forward, the cerebral arterial pulsation driven by the cardiac pump is strong enough to support efficient CSF flows, which is questionable (Aldea et al., 2019). Arguments have been put forward that vascular smooth muscle function is also considered important for CSF pressure and dynamics based on increased vascular smooth muscle reactivity modulating the clearance of intracranial metabolic waste (Cheng et al., 2019).

In addition, the circulation of CSF is also one of the factors affecting brain clearance system function. According to the “glymphatic system” hypothesis, CSF enters the brain *via* periarterial spaces, passes into the interstitium *via* perivascular astrocytic AQP4, and then drives the perivenous drainage of ISF and its solute (Plog and Nedergaard, 2018). Previous researches pointed out that this cerebral CSF circulation is only active during sleep or general anesthesia (Winsky-Sommerer et al., 2019). More and more evidence suggests that regular sleep time ensures the efficiency of the brain clearance system (Aguirre, 2016). Given the latest discoveries, the brain clearance system is closely related to neurodegenerative diseases, such as AD, improving sleep is an essential means of preventing neurodegenerative diseases (Albrecht and Ripperger, 2018). However, the effect of anesthesia on CSF circulation is still controversial (Taoka et al., 2018). So far, the most influential account of anesthesia on CSF circulation is found in the study by Gakuba et al. (2018), which used MRI and near-IR fluorescence imaging to investigate the impact of general anesthesia on the intracranial CSF circulation in mice. Contrary to what was initially expected, they found that CSF circulation was more active when awake while significantly impaired during general anesthesia, suggesting that the effects of anesthesia on the brain clearance system are related to the dose (Gakuba et al., 2018). Clinical studies on whether anesthesia can be used to regulate the function of the clearance system are still lacking.

## Blood-Brain Barrier

**FIGURE 3:**
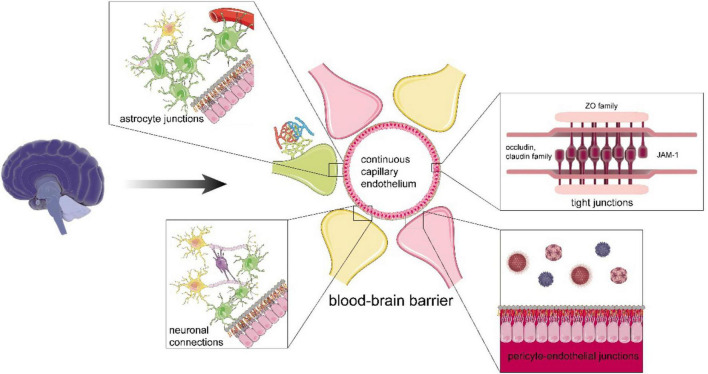
The functional structure of the blood-brain barrier (BBB). The BBB includes continuous capillary endothelium with tight junction (TJ), adherens junctions and gap junctions, pericyte-endothelial junctions, astrocyte junctions, neural connections, intact basement membrane, and glial membrane. The TJ is abundant in TJ proteins. For instance, claudin and scaffolding protein, such as zonula occludens family, form the backbone of TJ, occludin helps maintain the integrity and stability of TJ, and junctional adhesion molecule A mediates cell adhesion to restrict epithelial permeability. In terms of astrocytes, they can strengthen the BBB by junctions with ECs, communicate with myelinated nerve fiber and synapses, and support them. For neurons, their connections are enhanced by glial cells, such as oligodendrocytes, which can assist efficient jump transmission of bioelectrical signals and maintain the normal function of neurons.

The BBB cooperates with the glymphatic system in waste clearance and plays a dominant role in separating blood cells, exogenous pathogens, and circulatory wastes from the brain to maintain brain homeostasis (McConnell et al., 2017; Sweeney et al., 2019; Ueno et al., 2019) (Figure 3). With carrier-mediated transport on epithelium performing waste efflux and the clearance of its cellular components, BBB prevents CNS from accumulating metabolites and xenobiotics (Ueno et al., 2019). It is acknowledged that BBB can be divided into a basic functional unit called neurovascular unit (NVU), which consists of neurons, vascular cells, such as EC, pericytes, and gliocytes, including astrocytes and microglia. Mounting evidence suggests that NVU regulates BBB permeability, cerebrovascular net function, and neurogenesis (Obermeier et al., 2016; Saint-Pol et al., 2020). Under pathological conditions, the dysfunction and breakdown of constituent cells and relevant structures, such as tight junction (TJ), will lead to neurological disorders and deficits. The “Blood-brain barrier” section will attempt to describe how the cellular components with their junctions realize barrier function in BBB and elimination of wastes in the clearance system.

## Tight Junction

The TJ is the most substantial supporting structure in BBB formed on continuous capillary endothelium (Van Itallie and Anderson, 2014; Tietz and Engelhardt, 2015). TJ has plenty of specifically marked transmembrane proteins, including the occludin and claudin family, which communicate with other intracellular scaffolding proteins, such as the zonula occludens family (Tietz and Engelhardt, 2015; McConnell et al., 2017; Abdullahi et al., 2018) (Figure 3). However, the TJ can also be passive and changeable for nutrients and metabolites. It has been demonstrated that TJ synergizing with adherens junctions acts as an adjuster to the diffusion of solutes in plasma (Tietz and Engelhardt, 2015). Researchers have identified that Aβ itself could downregulate claudin-5 and occludin levels at the posttranslational levels to regulate TJ function, thereby promoting the efficiency of brain turnout and clearance of Aβ *via* the paracellular pathway through transendothelial cell receptors (Keaney et al., 2015). Hence, an appropriate opening of the TJ may promote the values of clearance of CSF and ISF through perivascular gaps. For example, Zn + highly accumulated in synapses can help open the TJ reversibly *via* the Glycogen synthase kinase-3 signaling (GSK3β)/snail-mediated signal transduction, i.e., the opening facilitates expelling macromolecular metabolites and toxins out of the brain cleaned (Xiao et al., 2018).

Under pathological conditions, the structure of TJ can be damaged, causing BBB to degrade even break down. Reactive oxygen species (ROS) may affect the integrity of TJ through the degradation of occludin, as evidenced by the elevated levels of occludin phosphorylation caused by ROS hydrogen peroxide (H_2_O_2_) at low concentrations, and occludin cleavage caused by H_2_O_2_ at moderate-to-high concentrations (Lischper et al., 2010). Neurons may thus be subjected to exogenous toxicants (Blanchette and Daneman, 2015). Besides, the TJ leakage can also result in the solute heaped up in vessels and tissues, hindering the drainage of ISF and CSF and causing ischemic lesions, such as ischemic stroke (Abdullahi et al., 2018). From the above mentioned text, opening the TJ can be helpful to deliver drugs targeted at brain parenchyma. Drugs targeting TJ-associated proteins, such as borneol, bradykinin, and hyperosmotic mannitol, and approaches, such as focused ultrasound, have been proven effective in opening TJ currently (Duan et al., 2016; Gonzalez-Mariscal et al., 2016).

## Cellular Components of Clearance System in the Brain

The glial cell is the executor of the function of the clearance system in the brain. As more adequately correlate with functional status, the “Vascular EC” section involves physiological fluctuations and pathological shifts, which can provide perspective guidance and meaningful solutions of diseases caused by the accumulation of metabolic waste products and neurotoxins in the brain in the future.

## Vascular Endothelial Cell

Forming blood vessels by chimerism and connection with each other, vascular ECs build a core barrier between brain parenchyma and external environment, with the assistance of their junctions, such as TJ. Besides the physical function in BBB mentioned earlier, vascular ECs also provide a selective passage for the import of macromolecular nutrients and circulating proteins and the efflux of toxins and metabolites (McConnell et al., 2017). Carrier-mediated transporters, such as nucleotide, hormone, and amino acid transporters, and receptor-mediated transporters, such as insulin and lipoprotein receptors, ATP-binding cassette (ABC), and ion transport channels, are all endothelium-dependent, regulating the homeostasis of the material delivery (Sweeney et al., 2019; Shubbar and Penny, 2020). For example, ABC family, such as ABCB1 and P-glycoprotein, on the endothelium has been proved effective in the removal of Aβ out of the brain (Pan et al., 2020; Shubbar and Penny, 2020). Factors, such as gene mutation, that can alter the phenotype of vascular ECs may result in the diminishment or disappearance of the waste clearance function (Oikari et al., 2020). In further research, the specific relationship between EC function-related gene mutation and disease still needs to be clarified, benefiting the symptomatic treatment of specific neurological disorders (Oikari et al., 2020). What is more, the upregulated expression of translocators on ECs and induced release of endothelial relaxation factor, such as NO may also offer a possibility to accelerate the flow of CSF and ISF in PVS, enhancing the function of the clearance system in the brain consequently (Cheng and Haorah, 2019).

## Astrocyte

Astrocytes, the coadjutant to build BBB with their endfeet attached to the vascular interface, also function as a doorman to control and clear CSF flow from paravascular spaces (Albrecht and Ripperger, 2018). In the last few years, more researchers shift their sights to the clearance of metabolic wastes and exogenous toxins that astrocytes perform, during which AQP4 takes a critical part. The AQP4 helps to remold to promote synaptic and vascular plasticity and assist astrocytes in eliminating dendritic spines (Valenza et al., 2019). In the clearance system, the AQP4 functions as a circulator participating in regulating the diameter of brain capillaries to guarantee interstitial flow and accelerate ISF circulation. It has been demonstrated that the polarized localization of AQP4 to perivascular endfeet can accelerate the CSF from subarachnoid to flow to brain interstitium in a convective bulk way, helping the glymphatic system clear the ISF and CSF simultaneously (Yang et al., 2017; Valenza et al., 2019; Zhang et al., 2020). Suppose the AQP4 localization has been altered or lost. In that case, astrocytes tend to release VEGFs and matrix metalloproteinases (MMPs) to increase BBB permeability, which will result in the accelerating process of BBB disruption and the deposition of insoluble Aβ along with subsequent reactive gliosis (Michinaga and Koyama, 2019; Valenza et al., 2019).

Interestingly, researchers also find a positive correlation between age and the expression of AQP4, especially in neurodegenerative disorders (such as AD) (Denver et al., 2019). Therefore, the downregulation and the fine control of the AQP4 show potential in the healing and prognosis of these diseases. Studies have shown that drugs, such as zonisamide and topiramate, can inhibit the function of AQP4. At the same time, the synthetic antibody to AQP4 also benefits a great deal in diseases caused by the autoantibody to AQP4, such as neuromyelitis optica (Verkman et al., 2017).

From the perspective of regulating BBB structure, astrocytes are also the hinge cable between BBB and the glymphatic system. As far as can be determined, consciousness and body posture affects the clearance rate of brain waste, in which sleep is a stimulating factor (Natale et al., 2021). While asleep, astrocytes may increase the interstitial volume by shrinking themselves, promoting ISF flow and the transformation between CSF and ISF. Also through this process, the glymphatic clearance is straightly facilitated, and the scavenging efficiency of BBB is also indirectly improved by increasing the flow of interstitial solute (Mendelsohn and Larrick, 2013; Verheggen et al., 2018; Zhang et al., 2021). Accordingly, BBB will also upregulate the activity of efflux transporters, such as ABCB1, under the control of the circadian clock (Zhang et al., 2021). Consequently, the BBB and the glymphatic system can cooperate and influence each other.

## Microglia

Microglia are resident cells in the innate immune system performing as CNS parenchymal macrophages, taking charge of digesting and degrading endogenous wastes in the brain, such as cellular debris, to accomplish neuronal and synaptic surveillance, developmental neuronal apoptosis clearance, and dendritic spine pruning (Casano et al., 2016; Dudvarski Stankovic et al., 2016; McConnell et al., 2017). Once microglia are exposed to declining and apoptotic cells in the very first stage of apoptosis, the expression of clearance-related genes, such as PRC2, is promoted, launching a series of clearance reactions in the very first stage of apoptosis (Ayata et al., 2018). Besides, microglia tend to share mutual support and promote phagocytosis, especially the one young microglia give to the older (Daria et al., 2017). Furthermore, microglia can be divided into pro-inflammatory (M1-like) phenotypes to immunosuppressive (M2-like) phenotypes according to the degree of activation at present (Loane and Kumar, 2016). Suppose microglia are aberrantly activated, which is common in acute CNS injury and neurodegenerative diseases. In that case, they can shift to M1-like phenotype, which results in the M1/M2-like polarization, and can release relative cytokines to speed up the disruption of BBB, consequently exacerbating the destruction of vessels and neurons (Loane and Kumar, 2016; Hammond et al., 2018; Hickman et al., 2018).

Moreover, their increased intrinsic function of phagocytosis and elimination can also damage neuron morphology and interconnection with others. Hence, it has been suggested that the detection of regions where the M1-like phenotype of microglia proliferate becomes increasingly significant in diagnosing neurodevelopmental disorders, such as autistic spectrum disorders, most of which are characterized by microglioma (Hammond et al., 2018). However, microglial activation also has several serious drawbacks, such as transferring microtubule-associated protein tau *via* exosomes in the brain and failing to clear the toxic metabolite (Asai et al., 2015; Li and Barres, 2018). Altogether, the mechanism of microglia causing aberrance and dysfunction of neurons in over-clearance circumstances remains to be elucidated, and the moderate regulation of microglia activation also needs to be explored (Dudvarski Stankovic et al., 2016). In the next step of research, discovering biomarkers of the M1-like phenotype microglia can be a target, which is beneficial in prompt diagnoses and treatment of neurodevelopmental disorders by more advanced pathological examinations, such as neuroimaging method.

## Pericyte

Pericytes are a group of cells embedded in the basement membrane of capillary ECs in the vasculature, act as ancillary cells to strengthen BBB, and assist functional members in the clearance system. As a member of NVU collaborates with astrocytes, microglia, and other neurons, pericytes participate in vascular development, permeability, and remodeling. Currently, pericytes have been proved to have the ability to differentiate into the neural lineage, contributing to the neurogenesis and inflammatory factor clearance after the brain injury or the chronic neuroinflammation, such as glioblastomas (Birbrair et al., 2017; Andreotti et al., 2018; Santos et al., 2019). In addition, pericytes reach to regulate the blood flow and interstitial flow of capillaries, modulating the clearance values of ISF and CSF (Burdyga and Borysova, 2018). Under pathological conditions, degenerated pericytes can directly decrease the pericyte-glia interface at BBB and then disrupt BBB (Giannoni et al., 2018; Klement et al., 2019). Consequently, it allows blood-derived toxins, such as autoantibodies murine immunoglobulin G (mIgG), to invade and then deposit in the brain parenchyma, leading to the diminishing clearance of gliocytes (Villasenor et al., 2017; Santos et al., 2019). Although the specific functions and behaviors of various subtypes of pericytes displayed in different stages of life in humans are not clear currently, the possibility and potential pericytes that have shown in TJ opening and blood flow control are undoubted, which provides us with a novel idea in the therapeutic measures of relative neurodegenerative diseases, such as AD (Sweeney et al., 2016).

The BBB and the glymphatic system have in common that they all aim to remove waste from CSF and ISF in the brain and reach mutual promotion and cooperation (Verheggen et al., 2018). Nevertheless, they also differ in the specific way of performing the function on account of structure. For BBB, barriers formed by cells and tight connections and selection of channels for substances, such as proteins, are the primary way to execute clearance. For the glymphatic system, however, it mainly separates waste from circulating CSF by promoting the convective movement of CSF between PVS and interstitial space to achieve the purpose of clearance (Abbott et al., 2018). On the one hand, when the clearance of more metabolites or toxic substances of BBB transporters reaches operational saturation, it seems that the glymphatic system can undertake the scavenging effect. On the other hand, the activity of convection in the glymphatic system can also stimulate the enhanced responsiveness of the AQP4 water channel and promote the function of BBB, especially astrocytes (Kress et al., 2014). In this context, BBB is more susceptible to aggressive barrier destruction factors, such as trauma, the collapse of cell junctions, such as pericyte loss, and the internal lesion-like upregulation of lipoprotein receptors (Keaney et al., 2015; Kaur and Sharma, 2018; Wei et al., 2019). At the same time, the glymphatic system is more susceptible to interference with CSF convection factors, such as altered pulsations and changed vasomotor tone (Brown et al., 2018; Rasmussen et al., 2018; Shackleton et al., 2019). In this process, inflammation and sleep disturbances may be the common factors that destroy both and cause the malignant cycle.

The NVU, composed of ECs, glial cells, pericytes, and neuronal cells, functions as a whole beyond the narrow scope of BBB operation, as well as performs and enhances the function of the removal of brain waste through intercellular communication and interaction (McConnell et al., 2017). As the cerebral vasculature extends, the vascular surface cover transforms from smooth muscle cells and pia mater to astrocytic endfeet and pericytes, during which the components of the NVU act synergistically to regulate vascular permeability, neuroimmune responses, and cerebral blood flow (CBF) for the transport of substances and waste products. Astrocytes play a critical role in this process by acting as a communication relay for neurons and blood vessels (Lv et al., 2021). Astrocytes transmit neuronal responses to changes in the surrounding environment (e.g., metabolite accumulation and hypoxia) in the form of cellular electrical and chemical signals, which ultimately regulate cerebral vascular tone and CBF. The impairment of NVU structures, such as the absence of normal vascular covering components in the intratumoral vasculature, can lead to changes in vascular permeability and corresponding alterations in the selective permeability of nutrients, metabolites, and exogenous toxicants (Lv et al., 2021). Simultaneously, the excessive activation of astrocytes may allow overproduction of Aβ in patients with AD; the impairment of the EC-mediated transport system could lead to decreased efficiency of Aβ clearance and its deposition in axonal compartments (Olabarria et al., 2010; Muoio et al., 2014; De Bock et al., 2016).

## 4 Potential Therapeutic Strategies Targeting Defects of Clearance System in the Brain

Exploring the regulatory mechanism of deficiencies in the clearance system may help identify the precise therapeutic target for intracerebral hemorrhage (ICH). Notably, the multi-omics technologies have made significant achievements in the research of neurodegenerative diseases (Nativio et al., 2020; Petermann-Rocha et al., 2020). A comparative analysis of driving gene expression in the physiological and pathological states of the clearance system will establish preliminary evidence for a link between them (Supplementary Table 1). The application of systematic multiomics approaches to precision medicine and systems biology has great potential to improve the care of patients with dysfunction of clearance systems. Besides, the target gene identified by multiomics studies can potentially be used for drug repositioning in ICH, which is approved to be cheaper, quicker, and effective (Armando et al., 2020) (Supplementary Table 2). For example, Caffeine targeting the *PDE1C* that is related to brain metabolites has been approved by the FDA for the short-term treatment of apnea of prematurity. Given the same putative drivers of the clearance system, those may maintain clearance system stability *via* relaxing the vascular smooth muscle and alleviate some pathological conditions caused by the disturbed clearance system. At present, direct evidence linking disease-driven analyses and molecular network dynamics in pathological contexts is lacking. Further research in neurological disorders is the next essential step in confirming diseases-driven networks associated with clearance system, thus developing novel therapeutic strategies that are more pertinent and specific.

## Conclusion

This review set out to provide a systematic account of the clearance system in the brain. The findings indicate that the flow of CSF through the glymphatic system and meningeal lymphatic vessels is driven by smooth muscle and cerebral arterial pulsation, thus clearing toxic and metabolic waste, including small molecules and biomacromolecules. Notably, BBB, microglia, and astrocytes seem to play a significant role in maintaining the process. These findings have substantial implications for understanding the clearance of metabolic waste from the brain. When the balance of the process is disturbed, how it contributed to pathological changes in the brain lays the groundwork for future research into physiological homeostasis and pathophysiological responses within the brain.

## Author Contributions

All authors listed have made a substantial, direct, and intellectual contribution to the work, and approved it for publication.

## Conflict of Interest

The authors declare that the research was conducted in the absence of any commercial or financial relationships that could be construed as a potential conflict of interest.

## Publisher’s Note

All claims expressed in this article are solely those of the authors and do not necessarily represent those of their affiliated organizations, or those of the publisher, the editors and the reviewers. Any product that may be evaluated in this article, or claim that may be made by its manufacturer, is not guaranteed or endorsed by the publisher.
